# Growth factor and cytokine characterization of canine platelet lysate with variable leukocyte concentration, plasma content, and heat-sensitive proteins

**DOI:** 10.3389/fvets.2024.1408080

**Published:** 2024-07-12

**Authors:** Thainá Lunardon, Scarlett M. Sumner, Melikasadat Mollabashi, Nikolia Darzenta, Emily Davis, Maria C. Naskou

**Affiliations:** ^1^Department of Pathobiology, College of Veterinary Medicine, Auburn University, Auburn, AL, United States; ^2^Department of Clinical Sciences, College of Veterinary Medicine, Auburn University, Auburn, AL, United States; ^3^Scott-Ritchey Research Center, College of Veterinary Medicine, Auburn University, Auburn, AL, United States

**Keywords:** platelet lysate, growth factors, leukocyte concentration, plasma proteins, complement

## Abstract

**Background:**

Platelet lysate is an acellular platelet product containing factors released from secretory granules, including cytokines and growth factors. This study aimed to evaluate different centrifugation methods used to prepare canine platelet lysate with variable content of leukocytes, plasma, and heat-sensitive proteins.

**Methods:**

Whole blood was collected from six dogs and two double-spin preparation methods were used to generate the platelet-rich plasma with reduced (PRP) and high (L-PRP) concentration of leukocytes. A portion of both methods underwent plasma depletion via centrifugation and platelet lysate was generated via freeze–thaw cycles. A portion of the generated platelet lysate underwent complement inactivation via heat treatment. Growth factors (TGF-β1, VEGF, TNF-α, PDGF-BB, HGF) were quantified in all different platelet lysate preparations using ELISAs.

**Results:**

Both platelet-rich plasma preparations had a 6.7-fold increase in platelet concentration. White blood cell (WBC) concentration compared to whole blood increased 1.2-fold times in PRP and 1.9-fold times in L-PRP. Negligible concentrations of platelets, WBC, and hematocrit were identified in all lysate groups. Statistically significant differences were identified for PDGF, VEGF, and TNF-α, and not for TGF-β or HGF. No growth factor differences were noted between centrifugation methods. PDGF was significantly higher in platelet lysate that was plasma depleted. VEGF was significantly higher in heat-treated lysate groups. TNF-α concentrations were overall very low, though were noted to significantly increase following plasma depletion.

**Conclusion:**

These results support that growth factors and cytokine release can be affected by the platelet lysate preparation and processing.

## Introduction

Platelets are non-nucleated, discoid-shaped cells that contain three different types of secretory granules, including lysosomes, dense granules, and α-granules. Through these granules, platelets provide a magnitude of functions beyond hemostasis, including initiation and propagation of the inflammatory process (1), anti-inflammatory effects (2, 3), and analgesic potential (4). The α-granules are the largest and most abundant of the platelet granules (1), which contain and release cytokines and growth factors that affect tissue regeneration, alleviate inflammation, and influence anabolic processes responsible for the recruitment and activation of other inflammatory cells (5, 6). Platelet derived products also exhibit antimicrobial activity, though the mechanisms are poorly understood; complement and complement binding protein present in platelet α-granules may be responsible for its antimicrobial effects (1, 7).

Growth factors present in the α-granules such as platelet-derived growth factor (PDGF) and vascular endothelial growth factors (VEGF), are important modulators of the physiological process of wound healing (3, 8) since they can enhance tissue regeneration (9), mesenchymal cell recruitment (10), synthesis and deposition of extracellular matrix (ECM), and angiogenesis (9, 10). Thus, recent practices include the administration of recombinant growth factors for wound healing. However, the rising production cost as well as limited availability precludes the clinical use of such products (9). Recent efforts have been focused on the development of platelet-derived products as an economical source of growth factors as well as cytokines, chemokines, and osteoconductive proteins for wound healing and tissue regeneration (8, 11–13).

Platelet lysate is a platelet-derived acellular product in which the platelet-derived growth factors have been released, the cell membranes removed, and thus the product can be stored for a prolonged period while offering the same benefits as platelet concentrates (14). Additionally, lysate allows wider allogeneic use, longer storage options, and can be pooled from different donors to alleviate individual donor-to-donor variability and allow optimal standardization (14–16). Platelet lysate can be generated from platelet concentrates via different methods such as plateletpheresis and manual centrifugation, including buffy-coat-based and tube method (13, 17), and via several commercially available systems that employ centrifugation or filtration-based methods (18–20). The activation of platelets leading to the release of their granule content can be achieved with freeze/thaw cycles, thrombin or calcium chloride (21, 22), sonication at different frequencies, or solvent/detergent treatment (22).

The role of the leukocytes in the therapeutic efficiency of these products remains controversial. A few studies have shown that a high concentration of leukocytes in platelet-derived products has been linked to impeding wound healing and tissue regeneration, increased scar tissue, and collagen degradation *ex vivo* (8, 23, 24). Additionally, the presence of pro-inflammatory mediators such as neutral proteases and acid hydrolases in the leukocytes, and thromboxane release from platelets may impede tissue regeneration (23). On the contrary, other studies have suggested that increased leukocyte content can increase growth factor secretion (3, 25, 26), and potentially contribute to bacterial killing of contaminated wounds via the release of myeloperoxidase (27).

Other constituents within the plasma of platelet products, such as fibrin and fibrinogen, may also influence its therapeutic effects. Heat treatment of human platelet pellet lysate, that is manufactured by the lysis of isolated platelets and is plasma free, has been studied to remove fibrinogen and procoagulant and proteolytic enzymes, including complement inactivation, as well as to ensure biocompatibility and safety. Fibrin and fibrinogen have a detrimental role in the modulation of acute inflammation (28) and can dose-dependently increase mesenchymal stromal cells secretion of pro-inflammatory cytokines, including MCP-1, IL-8, and IL-6. Fibrinogen and its cleavage products are known for their capability of altering vasoconstriction, angiogenesis, cell migration and proliferation in fibroblasts, smooth muscle cells, and lymphocytes (29). The prolonged accumulation of fibrin in injured tissue can result in significant outcomes, including persistent inflammation that may contribute to the formation of scar tissue and ulceration (28).

There are, however, no studies directly evaluating two commonly used manual centrifugation methods (pure platelet lysate [leukocyte reduced] versus leukocyte rich method with leukocyte reduced versus leukocyte rich) for the generation of canine platelet derived products and their different effects on growth factor and cytokine release. The objective of this study was to evaluate how different centrifugation methods used to prepare platelet derived products with variable leukocyte content, presence or absence of plasma proteins and heat-sensitive proteins (complement) affects final growth and cytokine concentrations. We hypothesized that the concentration of leukocytes, presence of plasma proteins and complement will affect the growth factor and cytokine concentration of canine platelet lysate.

## Materials and methods

### Blood acquisition

Six purpose bred canine blood donors (three male and three female beagles) aged 5.5–11.8 years old (median: 7.9 years), and body weights ranging from 13.5–38.8 kg (median: 19.40 kg) were included in this study. The health status of the donor dogs was evaluated prior to blood collection by clinical examination and evaluation of the hematocrit and plasma protein concentration. The study was approved by Auburn University Animal Care and Use Program (IACUC).

Whole blood was withdrawn aseptically from the jugular vein using a 22 g needle. A total of 100 mL of whole blood was collected from each donor and was divided into 8 mL blood collection tubes containing acid citrate dextrose-A solution (ACD-A; 3.2%). A small portion of blood was collected into an EDTA tube and a serum tube without a clot activator to perform a complete blood count (CBC) and serum biochemistry profile, respectively. The CBC was performed using ADVIA 2120 Hematology Analyzer (Siemens Medical Solutions, Pennsylvania, USA) and chemistry using Cobas C311 (Roche, IN, USA) at the Clinical Pathology Laboratory at Auburn University immediately after the collection. A blood smear of each donor was evaluated to confirm the results and the presence of platelet clumping.

### Generation of platelet-rich plasma

Whole blood was divided into 10 mL aliquots that were centrifuged in 15 mL conical tubes. Blood from each donor was separated equally to generate platelet-rich plasma via two different methods to create leukocyte-reduced (PRP) and leukocyte-rich (L-PRP) products. A double-spin preparation method was used for both methods.

**Figure 1:**
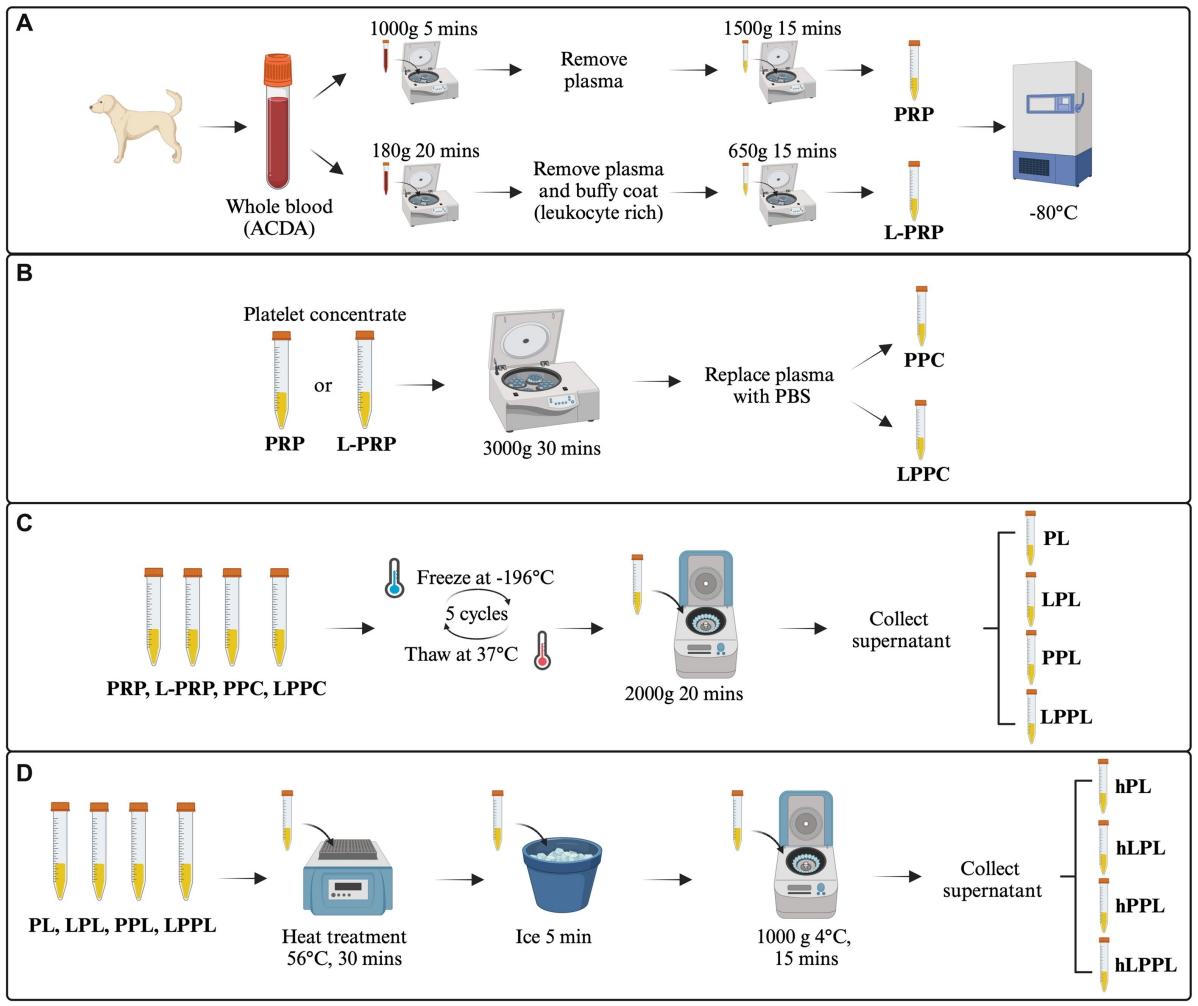
Overview of the study design. The image represents the experimental design and the procedures performed. **(A)** Generation of leukocyte-reduced platelet-rich plasma (PRP) and leukocyte-rich platelet-rich plasma (L-PRP). **(B)** Plasma depletion and generation of pure platelet pellet concentrate (PPC) and leukocyte rich platelet pellet concentrate (LPPC). **(C)** Generation of variable lysate formulations from PRP, L-PRP, PPC and LPPC (PL, LPL, PPL, LPPL). **(D)** Heat treatment to generate complement inactivated formulations from PL, LPL, PPL and LPPL (hPL, hLPL, hPPL, and hLPPL). ACD-A, acid citrate dextrose-A solution; PRP, leukocyte-reduced platelet-rich plasma; L-PRP, leukocyte-rich platelet-rich plasma; PL, leukocyte-reduced platelet lysate; LPL, leukocyte-rich platelet lysate; PPL, leukocyte-reduced platelet pellet lysate; LPPL, leukocyte-rich platelet pellet lysate; hPL, heat-treated PL; hLPL, heat-treated LPL; hPPL, heat-treated PPL; hLPPL, heat-treated LPPL. Created with BioRender.com.

PRP, designed to have a reduced leukocyte count, was manufactured as previously described (8) (Figure 1A). Briefly, blood was centrifuged at 1,000 g for 5 min without a centrifugal break at room temperature (Sorvall X Pro/ST Plus, ThermoFisher Scientific, USA). Following the first centrifugation, the whole plasma fraction above the buffy coat was pooled and centrifuged again at 1500 g for 15 min with a centrifugation break.

L-PRP, designed to have a high leukocyte count, was manufactured as previously described (30) (Figure 1A). Blood was centrifuged at 180 g for 20 min without a centrifugal break at room temperature. Subsequently, the plasma fraction and buffy coat layer were pooled from each tube and centrifuged at 650 g for 15 min with a centrifugation break to produce a platelet pellet.

After the second centrifugation for both methods, the platelet-poor fraction was removed and saved in a separate tube while the platelet concentration was determined via CBC (Heska Element HT5, Heska, Colorado, USA). Subsequently, the platelet-rich plasma preparations were resuspended with the appropriate amount of platelet poor plasma to achieve a final concentration of 0.8–1 × 10^6^/μL, as recommended by the American Red Cross for PRP (31). An aliquot from each preparation was submitted for a chemistry profile included concentration of bicarbonate, sodium, potassium, chloride, anion gap, calcium, glucose, total protein, and albumin.

### Plasma depletion of PRP

A portion of both PRP and L-PRP that were generated as above underwent plasma depletion to remove plasma-related proteins. Specifically, PRP and L-PRP were centrifuged at 3000 g for 30 min (Fresco 17 Microcentrifuge, ThermoFisher Scientific, USA) at 22°C. The plasma was removed, saved, and the platelet pellet was gently resuspended with an equal volume of sterile phosphate-buffered saline (PBS) to produce plasma-free leukocyte-reduced platelet pellet concentrate (PPC) and leukocyte-rich platelet pellet concentrate (LPPC) (Figure 1B).

### Generation of platelet lysates

Lysis of platelets from all preparations (PRP, L-PRP, PPC, LPPC) was performed by five freeze/thaw cycles in liquid nitrogen and thawing at 37°C. Following, all preparations were centrifuged at 20,000 g for 20 min to remove platelet membranes and other cellular debris (Figure 1C). This process produced leukocyte-reduced platelet lysate (PL), leukocyte-rich platelet lysate (LPL), leukocyte-reduced platelet pellet lysate (PPL), and leukocyte-rich platelet pellet lysate (LPPL). Sample supernatants were stored frozen at −80°C until further use. To decrease individual variability, lysates within each group were pooled from three donors to generate two batches of platelet lysate per method.

### Heat-treatment

A portion of pooled PL, LPL, PPL, and LPPL underwent heat treatment for complement inactivation in a dry bath at 56°C for 30 min as previously described (28). After heat treatment, the samples were cooled on ice for at least 5 min and then centrifuged at 10,000 g for 15 min at 4°C to remove any insoluble components. This process produced the following groups: heat-treated leukocyte-reduced platelet lysate (hPL), heat-treated leukocyte-rich platelet lysate (hLPL), heat-treated leukocyte-reduced platelet pellet lysate (hPPL), and heat-treated leukocyte-rich platelet pellet lysate (hLPPL) (Figure 1D). Sample supernatants were stored frozen at −80°C until further use.

### Growth factor and cytokine concentration

Growth factors and cytokines were quantified for all the above formulations using enzyme-linked immunosorbent assays (ELISAs), which were previously validated for use with canine plasma, and included: transforming growth factor-beta 1 (TGF-β1) (Human, Mouse/Rat/Porcine/Canine TGF-β1 Quantikine ELISA Kit, R&D Systems, MN, USA) (14, 19, 25, 32) vascular endothelial growth factor (VEGF) (Canine VEGF Immunoassay, R&D Systems, Minneapolis, MN, USA) (32), tumor necrosis factor α (TNF-α) (Canine TNF-α Immunoassay, R&D System, Minneapolis, MN, USA) (19, 32), platelet-derived growth factor (PDGF-BB) (Canine PDGF-BB ELISA KIT, Invitrogen, ThermoFisher Scientific, CA, USA) (8, 14, 19, 32) and hepatocyte growth factor (HGF) (Canine HGF ELISA Kit, Invitrogen, ThermoFisher Scientific, CA, USA). ELISAs were performed in duplicates and absorbance was read on a BioTek Synergy H1 multimode reader (Agilent, CA, USA) with wavelength absorption and corrected per manufacturer’s instructions.

### Statistical analysis

All data were imported into a statistical analysis program (GraphPad Prism; Graphpad Software Inc. San Diego, CA, USA). Normality was assessed via the visual examination of histograms of the residual, normal plots of residuals, and by using the Shapiro-Wilks test. The equality of variances was assessed using Levene’s test and plotting residuals against the fitted value. Hypotheses were tested by t-test and repeated-measures analysis of variance (ANOVA) after confirming normal distribution. Statistical significance was assessed using a 2-away analysis of variance (ANOVA). Tukey’s test was used to adjust for multiple paired comparisons. For non-parametric data, the analysis was performed using a mixed effects model with fixed effects being the preparation and formulation of lysate assessed while the lysate lot will be held as a random effect. All statistical analysis was performed at *p* < 0.05 level of significance. Continuous data were summarized and reported as mean ± standard deviation. All sample analyses were performed in duplicates.

## Results

### Hematologic values

The mean concentration of platelets, white blood cells (WBC), and hematocrit (HCT) obtained from the whole blood and each platelet-rich plasma preparation are presented in Table 1. The mean (± SD) platelet concentration for whole blood was 394.3 ± 164.34 × 10^3^/μL, and the WBC concentration was 8.27 ± 1.67 ×10^3^/μL. Both PRP and L-PRP had a 6.7-fold increase of their platelet concentration (2,646.0 ± 751.18 × 10^3^/μL and 2,661 ± 1,150.89 × 10^3^/μL, respectively). The WBC concentration of PRP increased 1.2-fold times compared to whole blood (10.2 ± 6.69 ×10^3^/μL) while L-PRP had a 1.9-fold times increase (15.7 ± 17.65 × 10^3^/μL) compared to whole blood. The HCT was almost negligible for both PRP and L-PRP.

**Table 1:** Descriptive results of hematological data presented as mean ± standard deviation from whole blood, and the various platelet formulations (*n* = 6).

Hematology analysis			
Preparation	PLT (x10^3^/μL)	WBC (x10^3^/μL)	HTC (%)
Whole blood	394.3 ± 164.34	8.27 ± 1.67	51.33 ± 3.73
Leukocyte-reduced platelet products			
PRP	2,646 ± 751.18	10.2 ± 6.69	0.40 ± 0.98
PPC	71.67 ± 63.09	0.04 ± 0.04	0.09 ± 0.16
PL	9.50 ± 6.36	0.03 ± 0.03	0 ± 0
PPL	1.5 ± 0.71	0 ± 0	0 ± 0
hPL	6.5 ± 4.95	0.03 ± 0.03	0 ± 0
hPPL	2.50 ± 2.12	0.01 ± 0	0 ± 0
Leukocyte-rich platelet products			
L-PRP	2,661 ± 1150.89	15.7 ± 17.65	1.80 ± 2.87
LPPC	158.0 ± 74.97	0.09 ± 0.16	0 ± 0
LPL	12 ± 1.41	0.01 ± 0	0 ± 0
LPPL	3.50 ± 3.54	0.01 ± 0	0 ± 0
hLPL	8 ± 7.07	0.06 ± 0.08	0 ± 0
hLPPL	1 ± 1.41	0 ± 0	0 ± 0

PLT, platelet concentration; WBC, white blood cell concentration; HTC, hematocrit; PRP, leukocyte-reduced platelet-rich plasma; PPC, leukocyte-reduced platelet pellet concentrate; PL, leukocyte-reduced platelet lysate; PPL, leukocyte-reduced platelet pellet lysate; hPL, heat treated PL; hPPL, heat treated PPL; L-PRP, leukocyte-rich platelet-rich plasma; LPPC, leukocyte-rich platelet pellet concentration; LPL, leukocyte-rich platelet lysate; LPPL, leukocyte-rich platelet pellet lysate; hLPL, heat treated LPL; hLPPL, heat treated LPPL.

Negligible concentrations of platelets, WBC and HCT were identified in all lysate formulations (PL, LPL, PPL, and LPPL).

### Chemical analysis

The mean concentration of the chemical components analyzed are reported in Table 2. The concentration of bicarbonate, chloride, calcium, total protein, and albumin were statistically significantly lower in both PRP and L-PRP compared to whole blood (Table 1). To the contrary, sodium and glucose concentrations were statistically significantly higher in PRP and L-PRP compared to whole blood.

**Table 2:** Descriptive results of chemistry analyzed data presented as mean ± standard deviation from whole blood, leukocyte-reduced platelet-rich plasma, and leukocyte-rich platelet-rich plasma (*n* = 6).

Chemistry analysis of platelet rich plasma			
Variable	WB	PRP	L-PRP
Bicarbonate (mmol/L)	22.47 ± 2.12	5.78 ± 1.82*	6.72 ± 1.81*
Sodium (mmol/L)	145.67 ± 1.37	169.83 ± 3.19*	170.17 ± 2.64*
Potassium (mmol/L)	4.05 ± 0.21	5.45 ± 1.52	4.68 ± 1.14
Chloride (mmol/L)	107.83 ± 1.94	60.0 ± 0*	60.0 ± 0*
Calcium (mg/dL)	11.00 ± 0.51	7.43 ± 0.42*	7.63 ± 0.44*
Glucose (mg/dL)	87.00 ± 12.76	712.50 ± 46.76*	691.17 ± 25.71*
Total Protein (g/dL)	6.47 ± 0.44	4.98 ± 0.41*	4.90 ± 0.29*
Albumin (g/dL)	3.69 ± 0.36	2.91 ± 0.27*	2.93 ± 0.23*

PLT, platelet concentration; WBC, white blood cell concentration; HTC, hematocrit; PRP, leukocyte-reduced platelet-rich plasma; L-PRP, leukocyte-rich platelet-rich plasma. *Denotes a significant difference compared to whole blood.

### Growth factor and cytokine concentrration

Overall, statistically significant differences among lysate groups were identified for the concentration of PDGF, VEGF, and TNF-α, while no statistically significant difference was identified for TGF-β and HGF concentrations.

**Figure 2:**
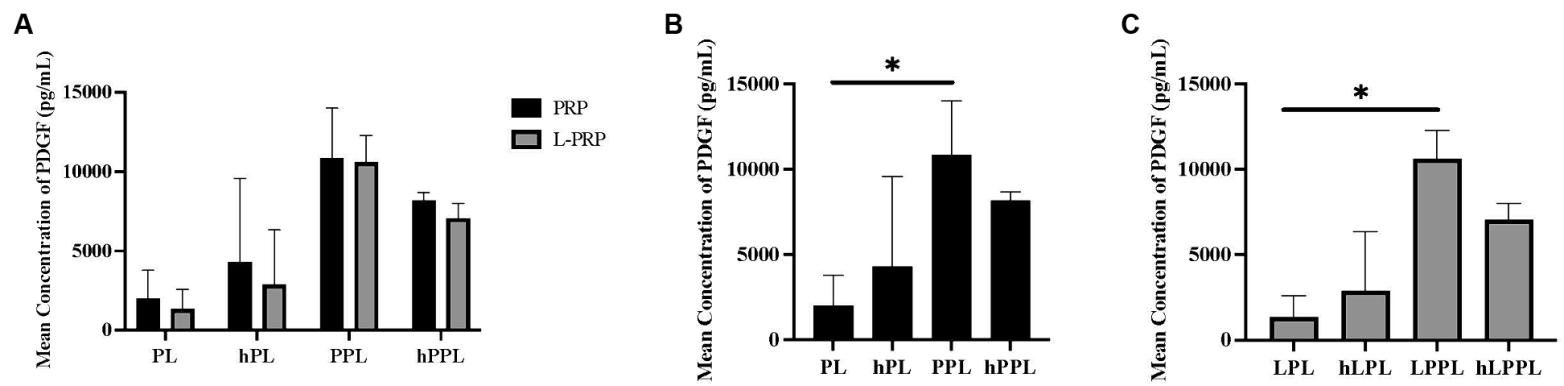
Mean concentration of platelet-derived growth factor (PDGF) between different preparations methods of lysate from both leukocyte-reduced platelet-rich plasma (PRP) or leukocyte-rich platelet-rich plasma (L-PRP). **(A)** Between variable lysate formulations generated from PRP **(B)**, or L-PRP **(C)** through ELISA (*n* = 2; mean ± SD). *p < 0.05. ELISA, enzyme-linked immunosorbent assay; PL, leukocyte-reduced platelet lysate; LPL, leukocyte-rich platelet lysate; PPL, leukocyte-reduced platelet pellet lysate; LPPL, leukocyte-rich platelet pellet lysate; hPL, heat-treated PL; hLPL, heat-treated LPL; hPPL, heat-treated PPL; hLPPL, heat-treated LPPL.

PDGF concentrations among the lysate groups are depicted in Figure 2. A statistically significant difference was not identified between all groups (Figure 2A). When separated by the centrifugation method, a statistically significantly higher concentration of PDGF was identified following plasma depletion for both platelet pellet lysate groups compared to platelet lysate groups. Specifically, PPL (10,599.6 ± 1676.48 pg/mL) and LPPL (10,841.9 ± 3177.03 pg/mL) had higher PDGF concentrations than PL (1988.00 ± 1792.94 pg/mL) and LPL (1349.83 ± 1231.06 pg/mL) (*p* = 0.043 and *p* = 0.037, respectively; Figures 2B,C). Nonsignificant trends noted for the concentration of PDGF included an increase following complement inactivation: hPL (4310.24 ± 5263.67 pg/mL) and hLPL (2882.76 ± 3443.67 pg/mL) tended to have higher PDGF when compared to PL and LPL. The opposite trend was observed in plasma depleted preparations following complement inactivation. A trend for a decreased PDGF concentration was noted in hPPL (8177.14 ± 494.18 pg/mL) and hLPPL (7055.34 ± 946.02 pg/mL) compared to PPL (10841.9 ± 3177.03 pg/mL) and LPPL (10599.6 ± 1676.48 pg/mL).

**Figure 3:**
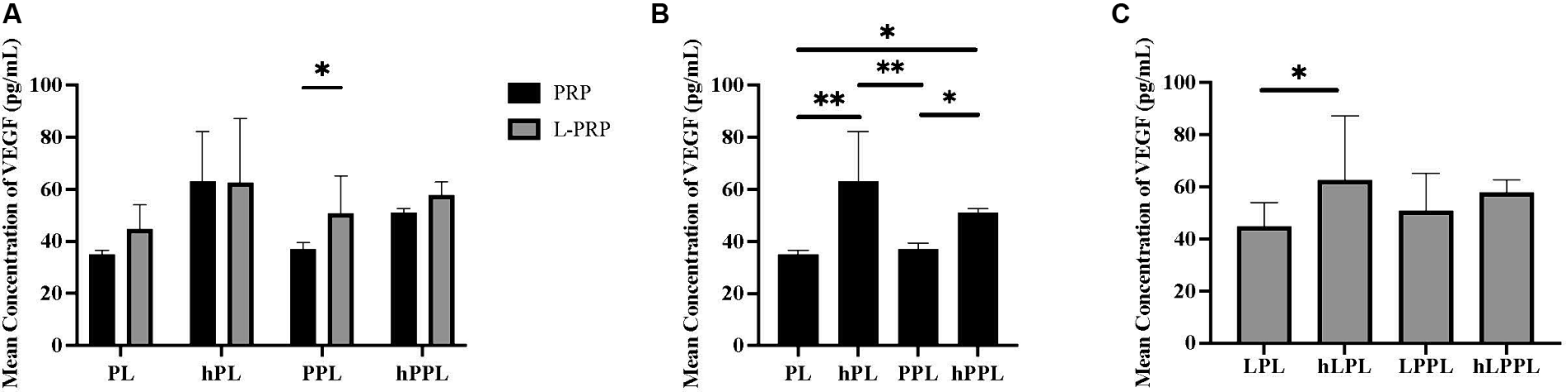
Quantification of vascular endothelial growth (VEGF) between different preparations methods of lysate from both leukocyte-reduced platelet-rich plasma (PRP) or leukocyte-rich platelet-rich plasma (L-PRP). **(A)** Between variable lysate formulations generated from PRP **(B)**, or L-PRP **(C)** through ELISA (*n* = 2; mean ± SD). *p < 0.05. **p < 0.01. ELISA, enzyme-linked immunosorbent assay; PL, leukocyte-reduced platelet lysate; LPL, leukocyte-rich platelet lysate; PPL, leukocyte-reduced platelet pellet lysate; LPPL, leukocyte-rich platelet pellet lysate; hPL, heat-treated PL; hLPL, heat-treated LPL; hPPL, heat-treated PPL; hLPPL, heat-treated LPPL.

VEGF concentrations are illustrated in Figure 3. VEGF concentrations were significantly higher in leukocyte-rich plasma depleted lysate (LPPL; 50.82 ± 14.23 pg/mL) compared to leukocyte-reduced (PPL; 37.08 ± 2.40 pg/mL, *p* = 0.480; Figure 3A), and in complement inactivated groups (hPL; 63.03 ± 13.57 pg/mL) compared to non-complement inactivated (PL; 35.00 ± 1.07 pg/mL, *p* = 0.006) and plasma depleted lysate (PPL; 37.08 ± 2.40 pg/mL, *p* = 0.008; Figure 3B). VEGF concentrations were significantly higher in plasma depleted and complement inactivated lysate (hPPL; 50.90 ± 1.68 pg/mL) compared to plasma depleted without heat inactivation (PPL; 37.08 ± 2.40 pg/mL, *p* = 0.047) and PL (35.00 ± 1.07 pg/mL, *p* = 0.032). Similarly, leukocyte-rich and plasma depleted lysate that was heat inactivated (hLPL; 62.57 ± 24.63 pg/mL) had significantly higher VEGF concentrations compared leukocyte-rich lysate (LPL; 44.80 ± 9.19 pg/mL, *p* = 0.024; Figure 3C). Overall, a trend was noted for VEGF concentrations to be higher in the leukocyte-rich preparation method, however, a statistically significant difference was not identified in groups other than LPPL and PPL as mentioned.

**Figure 4:**
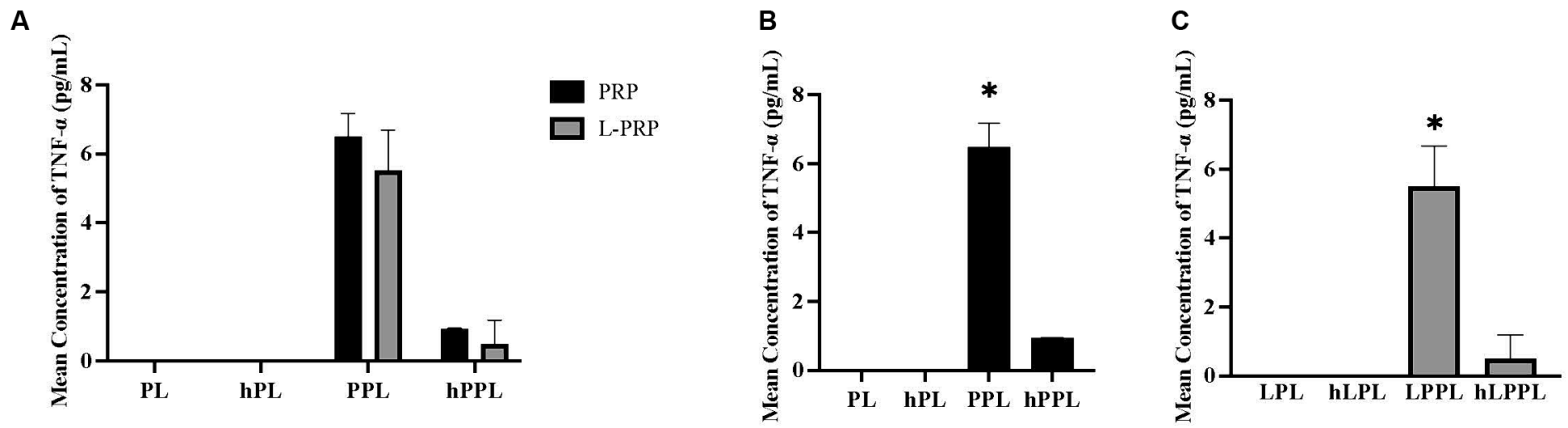
Quantification of tumor necrosis factor alpha (TNF-α) between different preparations methods of lysate from leukocyte-reduced platelet-rich plasma (PRP) or leukocyte-rich platelet-rich plasma (L-PRP). **(A)** Between variable lysate formulations generated from PRP **(B)**, or L-PRP **(C)** through ELISA (*n* = 2; mean ± SD). *p < 0.05 compared to the rest of the groups. ELISA, enzyme-linked immunosorbent assay; PL, leukocyte-reduced platelet lysate; LPL, leukocyte-rich platelet lysate; PPL, leukocyte-reduced platelet pellet lysate; LPPL, leukocyte-rich platelet pellet lysate; hPL, heat-treated PL; hLPL, heat-treated LPL; hPPL, heat-treated PPL; hLPPL, heat-treated LPPL.

TNF-α measurements are illustrated in Figure 4. Overall, the concentration of TNF-α was very low. No significant difference was noted when all groups were compared (Figure 4A). When groups were analyzed within each centrifugation method, significantly higher TNF-α concentrations were identified in plasma depleted lysate (PPL; 6.49 ± 0.68 pg/mL) compared to lysate (PL; below detection range, *p* = 0.012), complement inactivated lysate (hPL; below detection range, *p* = 0.012), and plasma depleted heat treated lysate (hPPL; below detection range, *p* = 0.018; Figure 4B). Similarly, significantly higher TNF-α concentrations were identified in the leukocyte-rich formulations that were plasma depleted (LPPL; 5.51 ± 1.18 pg/mL) compared to leukocyte-rich lysate (LPL; below detection range; *p* = 0.019), leukocyte-rich complement inactivated lysate (hLPL; below detection range, *p* = 0.019), and leukocyte-rich plasma depleted lysate that was heat treated (hLPPL;below detection range, *p* = 0.025; Figure 4C).

**Figure 5:**
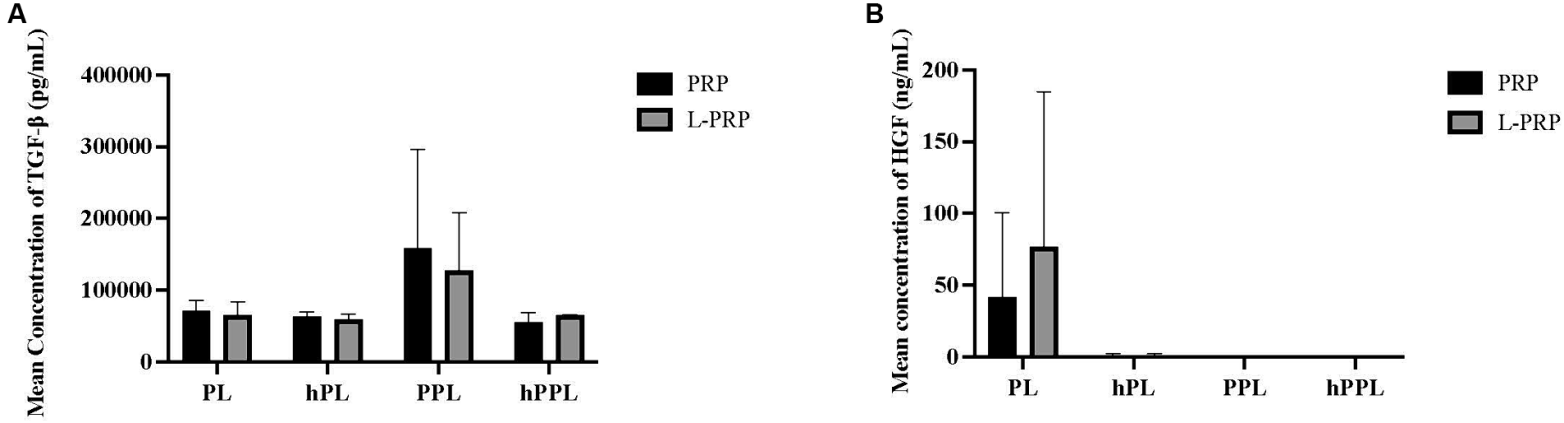
Quantification of transforming growth factor-β (TGF-β) **(A)** and hepatocyte growth factor (HGF) **(B)** from platelet rich plasma (PRP) or leukocyte and platelet rich plasma (L-PRP) as assessed through ELISA (*n* = 2; mean ± SD). ELISA, enzyme-linked immunosorbent assay; PL, leukocyte-reduced platelet lysate; LPL, leukocyte-rich platelet lysate; PPL, leukocyte-reduced platelet pellet lysate; LPPL, leukocyte-rich platelet pellet lysate; hPL, heat-treated PL; hLPL, heat-treated LPL; hPPL, heat-treated PPL; hLPPL, heat-treated LPPL.

TGF-β concentrations are shown in Figure 5A. A statistically significant difference was not identified between any of the groups. A trend toward higher TGF-β concentrations was noted in plasma depleted lysate (PPL; 158,048 ± 138,581 pg/mL) and leukocyte-rich plasma depleted lysate (LPPL; 127,067 ± 80984.8 pg/mL) compared to other groups, which decreased with heat/complement inactivation (hPL = 127,067 ± 80984.8 pg/mL, hLPPL = 54,887.7 ± 13,721 pg/mL).

HGF concentrations are presented in Figure 5B. No significant difference was noted between groups. Higher HGF concentrations were measured in lysate (PL; 41.7890 ± 58.4791 pg/mL) and leukocyte-rich lysate (LPL; 76.9850 ± 107.982 pg/mL) compared to the remaining groups, which had low to undetectable concentrations of HGF concentration.

## Discussion

In this study, we were able to show that platelet lysate produced by two different double-centrifugation methods followed by freeze/thaw cycles resulted in variable leukocyte concentration, while the presence or absence of plasma proteins, and complement or heat-sensitive factors affected growth factor and cytokine release.

In our study we used a double-spin preparation method for the generation of PRP and L-PRP. The purpose of the first centrifugation cycle is to separate the whole blood into three layers: the plasma, buffy coat, and red blood cell (13, 31) based on the cell’s density gradient (8). Platelets are the lightest cell, followed by the WBC, and lastly, the red blood cells which is the heaviest cell. However, the specific gravities of the cells slightly overlap, which limits the preparation of platelet concentrate free of other cell types by centrifugation method (8, 33). The second centrifugation is designated to separate platelets from plasma and further concentrate the platelets (8, 13). Therefore, the variation in the relative central centrifuge force (RCF) and time needs to be appropriate for the appropriate separation of the blood components and adequate platelet recovery (8).

The platelet count of the PRP in this study was similar to previously reported with a 6.7-fold increase compared to whole blood, though the leukocyte concentration was higher with a 1.2-fold increase in leukocyte count compared to whole blood (8). L-PRP also achieved a 6.7-fold increase in platelet concentration and a 1.9-fold increase in leukocyte count, a higher platelet concentration and a similar leukocyte concentration compared to previously described concentrations by Attili et al. (30). It should be noted that both centrifugation methods resulted in products characterized by an increased concentration of leukocytes compared to whole blood. The leukocyte-reduced method previously performed by Shin et al. noted a 2-fold reduction of leukocytes compared to whole blood in contrast to this study which noted a 1.2-fold increase in leukocyte concentration (8). Future studies should evaluate platelet products that are more effectively depleted of leukocytes compared to leukocyte-rich products. It should be noted, that platelet apheresis would be the ideal method of choice for the production of a completely leukocyte-reduced product (34).

According to the American Red Cross policy, platelet-rich plasma products are defined as those containing 1,000 × 10^3^ platelets μL^−1^ and accumulated data suggests that the platelet-rich plasma products must achieve a minimum platelet count of 300 × 10^3^ platelets μL^−1^ to exhibit a therapeutic effect (8, 13, 31). Moreover, in human medicine it is recommended that all platelet transfusion products have a leukocyte concentration less than 500,000 (13). In our study, both methods achieved the guidelines of The American Red Cross regarding the minimum and maximum concentration of platelets and leukocytes. Because both methods produce a high yield of platelets, the final platelet concentration was adjusted to 1,000 × 10^3^/uL for the rest of the comparisons.

Our chemical analysis showed statistically significant differences between both PRP and L-PRP and whole blood. It is likely that the release of granule contents and/or the presence of leukocytes are responsible for the observed changes. The presence of ACD-A as an anticoagulant, which is commonly used for the preparation of platelet-rich products, likely contributed to the chemical analyte variation compared to whole blood that was collected with EDTA as an anticoagulant.

Beyond centrifugation methods, this study investigated the effects of plasma removal and complement inactivation on growth factor and cytokine concentrations. It has been previously demonstrated that plasma removal allows for the depletion of detrimental factors for tissue regeneration, such as fibrinogen and procoagulant enzymes (28) via replacement of the plasma with PBS. Specifically, the presence of fibrinogen in platelet products can lead to excessive activation of the coagulation cascade and gel formation (33) and has a detrimental role in acute inflammation (28). Complement inactivation was pursued to further inactive proteolytic enzymes, remove pro-thrombotic factors and unwanted plasma proteins to prevent the side effects associated with toxicity caused by fibrinogen deposition (28, 35).

In this study, plasma removal resulted in highest concentration of PDGF-BB when measured in both PPL and LPPL compared to PL and LPL. The removal of plasma and replacement with PBS, which included an extra centrifugation cycle, may have caused further platelet degranulation and release of PDGF. Following complement inactivation, PDGF-BB concentrations had a nonsignificant trend to increase in hPL and hLPL compared to PL and LPL respectively, but decreased when complement was inactivated in hPPL and hLPPL derived products. While this was not a significant difference in this study, one previous study demonstrated a decreased PDGF-AB concentration following complement inactivation made from platelets only (PPL) (35). However, Chou et al. found no significant difference in PDGF-AB concentrations after complement inactivation of both PL and PPL (36).

On the other hand, heat treatment for complement inactivation resulted in a higher concentration of VEGF in both hPL and hPPL. A similar effect was noted for hLPL and hLPPL. This finding was in accordance with the study performed by Chou et al. (36) where higher concentrations of VEGF were identified following heat treatment of PPL. However, these results conflict with those previously reported in which a decreased concentration of VEGF was identified following heat treatment for complement inactivation in PPL (35). It is known that hypoxia stimulates the production of hypoxia-inducible factor 1ɑ (HIF-1ɑ), which can trigger the production of VEGF and other proangiogenic factors, including PDGF and TGF-β (37). It is possible that during our manufacturing process, a hypoxic environment was induced to the platelets resulting in a further release of VEGF. Another possibility is that growth factor analysis was performed in frozen samples, and it is possible that the plastic surface of the storage tubes had an effect on growth factor concentration. Moreover, an additive solution was not added to the products to improve stability, and this needs to be further investigated.

Studies conducted in human derived platelet products reported a higher concentration of growth factors such as PDGF, VEGF, TGF-β, and EGF in plasma depleted platelets products (PPL) (35, 36). In this study, this effect was encountered for PDGF, TNF-α, and TGF-β, but not for VEGF or HGF. Moreover, heat treatment for complement inactivation has been found to reduce the concentration of the above-mentioned growth factors except for TGF-β (35). Other studies did not encounter such an effect following heat treatment of the samples with the exception of HGF, fibroblast derived growth factor (FGF) and brain derived neurotrophic factor (BDNF) (36). Moreover, the above studies were performed in human derived platelet product, and it is possible that canine platelet characteristics and *in vitro* modification affects growth factor release differently.

The VEGF concentration in LPPL was significantly higher than PPL. It has been demonstrated that the presence of leukocytes can affect the release of growth factors from platelets (38). Castillo et al. found higher concentrations of PDGF-AB, PDGF-BB, and VEGF in leukocyte rich PRP compared to leukocyte poor PRP. However, the concentrations were not evaluated in plasma depleted products (26). Thus, it is possible that the presence of the leukocytes in the final product can affect growth factor release. However, note that the overall mean concentration of leukocytes between the two PRP methods did not strongly differ while plasma depletion has not previously evaluated (26). Future studies should focus on the production of platelet derived products with higher leukocyte concentration and the exact effect on growth factor and chemokine release from canine platelets.

TNF-α, which is an inflammatory cytokine, was evaluated to assess whether the mode of platelet lysate production might cause a spontaneous inflammatory process. Overall, the concentration of TNF-ɑ was very low among all groups. However, a statistically significantly higher TNF-α concentration was noted in PPL and LPPL compared to the rest of the groups. Heat inactivation resulting in a decreased concentration of TNF-α as seen in hPPL and hLPPL, but this difference was not statistically significant. It is worth mentioning that TNF-α concentrations were minimal or absent in lysate samples that were not further manipulated by plasma removal, while heat treatment did not seem to affect the concentration of TNF-α. Τhis is in accordance to previous studies that found that canine platelet products that did not undergo activation had minimal or absent concentrations of TNF-α (18). The degree of platelet activation was not quantified in this study and future studies should evaluate the expression of CD62P as a platelet activation marker.

Different modes of platelet lysate preparation produced variable concentrations of TGF- β with no statistical significance. However, a trend was noted for plasma depletion to increase the concentrations of TGF- β while following heat treatment decreased TGF-β concentrations. As mentioned above, a similar finding has been previously reported (35, 36) where TGF-β increased in plasma depleted products however both studies found an increased concentration of TGF-β following heat treatment (35, 36). Ιn this study, ELISA kits that detect activated TGF-β1 were used, and thus it is possible that heat treatment inhibited further platelet activation and/or accurate detection of the growth factor of interest via our method of detection. Furthermore, this study employed a freeze/thaw process for platelet activation, and no exogenous substances such as thrombin or calcium chloride were added to the products. The platelet activation method may have played a role in final growth factor concentration as well as its variation with additional processing such as plasma depletion and heat treatments. The process implemented for platelet lysis via a five freeze/thaw cycle procedure was recently shown to provide optimal release of growth factors (39). Most crucial, the freeze/thaw method eliminates the addition of any exogenous degranulation agents (36).

HGF is a mitogen for endothelial cells (40). Studies have shown that an anti-inflammatory function in tendon cells is mediated by HGF (2), and an anti-inflammatory function in chondrocytes is mediated by HGF and TNF-ɑ (3). In addition, HGF inhibits the release of pro-inflammatory cytokines and increases the release of IL-10 in LPS-activated macrophages (41). HGF was noted to be predominantly present in PL and LPL while a significant difference was not detected compared to other groups. A nonsignificant trend for HGF concentration to decreased following plasma depletion and heat treatment was noted. These findings are in accordance with those previously reported in the human literature (36) where HGF statistically decreased following heat treatment of both PL and PPL.

Just as plasma and platelets play an important role in coagulation, platelet derived products promote coagulation as well as the generation of cross-linked fibrin fibers, which is particularly important for soft tissue wound healing (42). However, this function is detrimental for other applications such as treatment for brain related disease or ocular disease (43). Thus, future studies should evaluate the effect of platelet lysate preparation on thrombin generation, thrombin proteolytic activity, presence of pro-coagulant molecules such as phosphatidylserine and activated factor XIa.

In summary, our results proved that growth factor and cytokine release can be affected by the mode of platelet lysate preparation and the presence of leukocytes in the final product. These differences can have a profound effect on the physiological and clinical function of platelet derived products and such information should be carefully evaluated when developing and selecting specific platelet lysate products for clinical applications.

## Data availability statement

The original contributions presented in the study are included in the article/supplementary material, further inquiries can be directed to the corresponding author.

## Ethics statement

The animal study was approved by Auburn University Institutional Animal Care and Use Committee (IACUC). The study was conducted in accordance with the local legislation and institutional requirements.

## Author contributions

TL: Data curation, Investigation, Methodology, Writing – original draft, Writing – review & editing. SS: Data curation, Investigation, Methodology, Project administration, Supervision, Writing – original draft, Writing – review & editing. MM: Data curation, Investigation, Writing – review & editing. ND: Data curation, Investigation, Writing – review & editing. ED: Data curation, Methodology, Supervision, Writing – review & editing. MN: Conceptualization, Data curation, Formal analysis, Funding acquisition, Investigation, Methodology, Project administration, Resources, Supervision, Validation, Visualization, Writing – original draft, Writing – review & editing.
